# Extended Active Space Ab Initio Ligand Field Theory: Applications to Transition-Metal Ions

**DOI:** 10.1021/acs.inorgchem.4c03893

**Published:** 2024-12-19

**Authors:** Shashank V. Rao, Dimitrios Maganas, Kantharuban Sivalingam, Mihail Atanasov, Frank Neese

**Affiliations:** † Max-Planck-Institut für Kohlenforschung, Kaiser-Wilhelm-Platz 1, Mülheim an der Ruhr 45470, Germany; ‡ Institute of General and Inorganic Chemistry, Bulgarian Academy of Sciences, Akad. Georgi Bontchev Street 11, Sofia 1113, Bulgaria

## Abstract

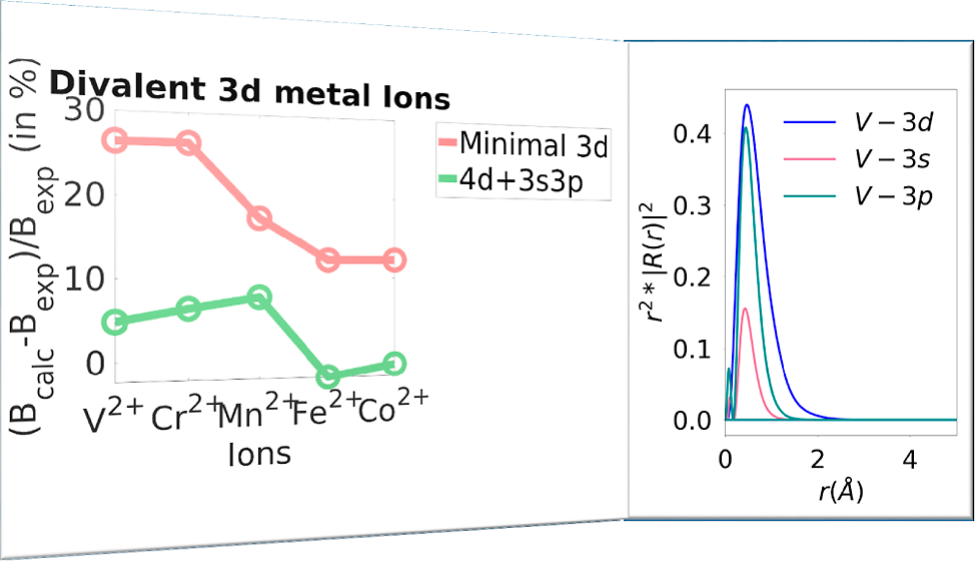

Ligand field theory (LFT) is one of the cornerstones of coordination chemistry since it provides a conceptual framework in which a great many properties of d- and f-element compounds can be discussed. While LFT serves as a powerful qualitative guide, it is not a tool for quantitative predictions on individual compounds since it incorporates semiempirical parameters that must be fitted to experiment. One way to connect the realms of first-principles electronic structure theory that has emerged as particularly powerful over the past decade is the ab initio ligand field theory (AILFT). The original formulation of this method involved the extraction of LFT parameters by fitting the ligand field Hamiltonian to a complete active space self-consistent field (CASSCF) Hamiltonian. The extraction was shown to be unique provided that the active space consists of 5/7 metal d/f-based molecular orbitals (MOs). Subsequent improvements have involved incorporating dynamical correlation using second-order N-electron valence state perturbation theory (NEVPT2) or second-order dynamical correlation dressed complete active space (DCDCAS). However, the limitation of past approaches is that the method requires a minimal space of 5/7 metal d- or f-based molecular orbitals. This leads to a number of limitations: (1) neglect of radial or semicore correlation would arise from the effect of a second d-shell or an sp-shell in the active space, (2) a more balanced description of metal–ligand bond covalency is lacking because the bonding ligand-based counterparts of the metal d/f orbitals are not in the active space. This usually leads to an exaggerated ionicity of the M–L bonds. In this work, we present an extended active space AILFT (esAILFT) that circumvents these limitations and is, in principle, applicable to arbitrary active spaces, as long as these contain the 5/7 metal d/f-based MOs as a subset. esAILFT was implemented in a development version of the ORCA software package. In order to help with the application of the new method, various criteria for active space extension were explored for 3d, 4d, and 5d transition-metal ions with varying charge. An interpretation of the trends in the Racah B parameter for these ions is also presented as a demonstration of the capabilities of esAILFT.

## Abstract 2

Ligand field theory (LFT) is one of the cornerstones of coordination chemistry since it provides a conceptual framework in which a great many properties of d- and f-element compounds can be discussed.

## 1 Introduction

In transition-metal chemistry, ligand field theory (LFT) has been a valuable model in explaining the relationship between a wide variety of experimental data from spectroscopy, magnetism, and so forth. This model has had notable successes, for example, explaining the heat of hydration of transition metals using the parameters derived from absorption spectroscopy,^26,29,49^ also in areas like magneto-structural correlation.^27,32,56,57^ LFT can be a beneficial tool for understanding the guiding principles behind a variety of chemical and physical trends in transition-metal complexes. One often observes crystal field theory (CFT) as distinguished from the ligand field theory. The difference is that CFT is based on a purely electrostatic interpretation, while LFT acknowledges that the metal d-orbitals are engaged in chemical bonds with the ligands. For the purposes of this work, the difference is mostly formal. Traditionally, fits to one or more experimental sources of data were used to obtain the parameters for the LFT Hamiltonian. However, these experimental fits can suffer from underdetermined equations, particularly when the complex has low symmetry. Such an underdetermined set of parameters can lead to nonunique fits that can potentially jeopardize the understanding of chemical behavior using the model.

Since the experimental fits are difficult or may suffer from being underdetermined, it is therefore desirable to develop theoretical methods that provide unique values for the LFT parameters. Such theoretical values may be used to study chemical trends or to provide excellent starting values for fits to experimental data. Unfortunately, there is no precise theoretical definition of the ligand field parameters in terms of ab initio electronic structure theory. In fact, if one calculates the LFT parameters as they come out of the model, highly unrealistic values will result, quite similar to the situation that is met with, for example, the resonance parameter in the Hückel theory of aromatic compounds. Hence, the connection between LFT and first-principles electronic structure theory must be achieved in a different way. Over the years, numerous attempts have been made to connect the results of either density functional theory (DFT) or wave function-based ab initio calculations to LFT parameters.^7−9,12,13,47^ While the different methods have met with various levels of success, they had the common feature that they did not lead to a unique extraction of the LFT model parameters. As discussed in detail previously,^10,55^ if one just fits, e.g., excitation energies, there are remaining ambiguities with respect to the way the LFT parameters are extracted.

A solution to the uniqueness of the extraction problem, that has met with considerable success, has been the introduction of the ab initio (AI) ligand field theory (AILFT).^11^ The central idea at the heart of the AILFT approach is that instead of fitting excitation energies, one simultaneously fits the entire ligand field Hamiltonian matrix to a suitable equivalent obtained by multireference wave function-based ab initio calculations. This requires the introduction of an ab initio-effective Hamiltonian that has a logical structure that is isomorphic with the structure of the LFT Hamiltonian. It has been found that, for a d^N^ problem, this effective Hamiltonian can be derived in terms of the complete active space configuration interaction (CAS-CI) matrix with N-electrons in five (d-elements) or seven (f-elements) molecular orbitals that must be dominantly based on the metal d- and f-shells to make sense. All that is then required is that the active orbitals are suitably canonicalized to be in a form where each many-particle basis function (MPBF), Slater determinants (DETS), or configuration state functions (CSFs)^10,20,21^ that enters the CASCI or the ligand field Hamiltonian matrix is constructed in the same order and in the exact same way. In this case, there is a 1:1 correspondence between the two matrices. It has then been shown that the extraction is unique because the ligand field Hamiltonian is linear in all of its parameters. In order to improve on the results of this straightforward recipe, the CASCI matrix can be dressed in various ways in order to introduce the effects of dynamic electron correlation.^35−37,50^ In the simplest approach, one can transform the CASCI matrix into the basis of its eigenstates and replace the diagonal energies with energies obtained with correlation methods, such as the N-electron valence perturbation theory to second-order (NEVPT2)^2−4^ or complete active space second-order perturbation theory (CASPT2).^1,33^ While these approaches lead to distinctive improvements in the extracted LFT parameters compared to empirical fits, the diagonal nature of the correction has limitations. Subsequently, it has been shown that the dynamic correlation-dressed complete active space method (DCD-CAS)^35−37,50^ or the Hermitian quasi-degenerate NEVPT2 variant (HQD-NEVPT2),^37^ both of which treat all matrix elements on equal footing, leads to improved and more balanced extractions.^35^ Since its introduction in 2012, the AILFT approach has seen many successful applications in d- and f-element chemistry,^27,56^ including systematic studies on lanthanides^5,31^ and actinides.^31^

There are, however, still significant limitations of the AILFT approach. The nature of the AILFT extraction requires the CASCI or effective Hamiltonian matrix to be of the same dimension as the LFT Hamiltonian. This means that an extension of the CASSCF calculation, with the active space larger than the d-space (or f-space for lanthanides) to incorporate static correlations without increasing the number of AILFT parameters, is not possible with the original recipes.

In this work, we provide a solution to this limitation by introducing another effective Hamiltonian^41,42^ that is based on extended active spaces in CASSCF calculations. This method (esAILFT) allows us to perform AILFT calculations on the basis of extended CASSCF active spaces to, in principle, any number of orbitals. The central idea is that esAILFT would allow for the inclusion of effects such as radial correlation of d-orbitals and reduce the overestimation of the ionic character in the CASSCF calculation. While esAILFT as presented here is general, we also benchmark various selection criteria for the extended active space to provide a guideline for the use of the esAILFT method. In particular, we analyze the effect on the Racah B parameter in transition-metal ions as a means to gauge the improvement offered by the present formalism over the original one. Chemical applications of esAILFT will be reported in future publications.

## 2 Theory

### 2.1 Ab Initio Ligand Field Theory

#### 2.1.1 Crystal- and Ligand Field Theory

The model of crystal field theory (CFT) consists of envisioning a central metal in a d^*n*^ electronic configuration that is perturbed by the electrostatic field created by the ligands that are modeled as point charges or point dipoles. CFT can be approached from two directions: (a) in the weak field approach, one starts from the Russell–Saunders multiplets of the free ion and studies how they evolve under the influence of the ligand field. (b) In the strong field approach, one first studies how the d-orbitals split under the influence of the ligand field before constructing many electron terms that are then allowed to interact via CI. If taken to completion, both approaches provide identical answers. A highly systematic treatment that leads from the weak-field to the strong-field limit has been provided by Tanabe and Sugano^22^ and is summarized in the famous Tanabe–Sugano diagrams that hold for cubic symmetry.

In CFT, the electron–electron repulsion is parametrized as in the free atom or ion. Two parameters are required to describe the splitting between different terms. They can either be taken as the Condon–Shortley parameters F_2_ and F_4_ or (more commonly) as the Racah parameters B and C. A third parameter F_0_ (Condon–Shortley) or A (Racah) affects all terms of a given d^*n*^ configuration in an identical manner and can be dropped. After adopting the commonly used approximation that *C* = 4*B*, the electron–electron repulsion can be reduced to a single parameter, B.

The electrostatic crystal field provides a one-particle perturbation. As such, it can be expressed on the basis of the five d-orbitals as a 5 × 5 potential matrix *V* that is real and Hermitian. This matrix contains information about the symmetry and strength of the crystal field. There are 5 × 6/2 = 15 independent parameters in matrix *V*. There are various ways to interpret these parameters. The electrostatic interpretation is only one of the possibilities. Another, equally valid and perhaps more chemically satisfying, way to parametrize the matrix V is the angular overlap model that was inspired by molecular orbital theory.^53^

For modeling magnetic properties, one final parameter is required: the one-electron spin–orbit coupling constant ζ. There are mathematical expressions that would seemingly allow one to calculate all CFT parameters as integrals over the metal d-orbitals.^25^ While this is readily doable in closed form, it would lead to very poor numerical results. It would also be, in our opinion, a misunderstanding of what CFT is aiming to achieve: CFT provides a conceptual framework in which the integrals serve as semiempirical parameters to be fitted to experiment. In these fits, the symmetry of the coordination environment must be respected. For example, in cubic symmetry, the 15 parameters in *V* are reduced to a single-fit parameter, 10Dq, the ligand field splitting. This physically appealing model captures the key physics at play in these systems and is the focus of the present work. Approaches that increase the number of parameters to the LFT model such as the “Trees correction” and beyond have also been analyzed in the literature^52,58,59^ and can have varying physical interpretabilities.

The known values of these parameters should be considered to be rough order of magnitude estimates that help guide any fit procedure into a physically reasonable solution. Quite typically, one observes that B and ζ need to be reduced from their atomic values in order to fit the experiment. This has been termed the “nephelauxetic effect.” The nephelauxetic effects received its name from the notion of a “cloud expansion,” implying that a larger d-orbital gives electrons more space to avoid each other and hence reduces the electron repulsion. However, the real reason for the observed reduction in the Racah parameters is more complicated and involves covalent dilution brought about by the formation of molecular orbitals (traditionally referred to as “symmetry-restricted covalency”^28^) together with complex changes in the radial distribution functions (traditionally referred to as “central field covalency”^28^). An in-depth discussion of these phenomena is outside the scope of this work and will be the focus of a future publication. The interpretation of this effect has been evergreen in the theory of transition-metal electronic structure.

#### 2.1.2 Ab Initio Ligand Field Theory

Ab initio ligand field theory was designed to act as a bridge between rigorous first-principles quantum chemical calculations and the model of CFT. Thus, its main mission is the extraction of unambiguous values of the parameters V–ζ using the ab initio electronic structure theory. In order to accomplish this task, a mapping is constructed between the many-particle functions of the ab initio theory and the many-particle functions that arise in the strong field limit of CFT/LFT.

In the original version of AILFT, the construction hinged on a CAS(*n*,5) active space that had five metal-d-based molecular orbitals in it. In order to identify those with the (fictitious) metal-d-orbitals that occur in LFT or CFT, some orbital preparation needs to be performed that brings the ab initio MOs into a standard order that matches the semiempirical theory. One way to achieve this is, for example, to diagonalize the  operator.

Once one has established a 1:1 correspondence between the ab initio MOs and the LFT orbitals, it is straightforward to construct the many-particle Hamiltonian in a consistent way. In the ab initio framework, the complete active space configuration interaction (CASCI) matrix is built by whatever systematic procedure is used to construct the configuration state functions of a given spin and space symmetry. The same approach is then used to construct strong-field configurations in the LFT Hamiltonian. The result is the ab initio as well as LFT full-CI matrices in the CAS(*n*,5) space.

The ligand field parameters V, B, C, and ζ are then found by a least-squares minimization that minimizes the difference between the ab initio and ligand field Hamiltonian matrices. Since the ligand field parameters all occur in a linear fashion in theory, this fit boils down to solving a linear equation system that either has no or a unique solution. Hence, the procedure provides unique values for the ligand field parameters. These can then be used in order to construct another layer of interpretation, for example, by interpreting the V-parameters in terms of the angular overlap model.

In mathematical terms, the ligand field Hamiltonian can be formally expressed as
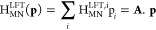
1where the parameter vector **p** includes the 15 elements representing one triangle of the one-electron matrix and the electronic repulsion parameters B and C (and potentially also ζ). It should be emphasized that eq 1 is general in nature.

Let us illustrate this abstract concept with a concrete example. A Ni^2+^ d^8^ ion in an octahedral (O_h_) ligand field coordination environment has ground-state electron configuration ^3^A_2g_ (t_2g_^6^e_g_^2^) with a total spin (*S* = 1). Considering single and double spin-conserving electron excitations, one may reach the following excited-state configurations ^3^T_1g_ + ^3^T_2g_ (t_2g_^5^e_g_^3^) and ^3^T_1g_ (t_2g_^4^e_g_^4^) of three triply degenerate excited states. This leads to an LFT Hamiltonian that is a block diagonal with a different block for each of these states. Since there is more than one configuration that leads to ^3^T_1g_, the block corresponding to it is a 2 × 2 symmetric matrix. Thus, we have five nonzero matrix elements of the full ligand field Hamiltonian which can be expressed in terms of the Racah parameters and the one-electron parameters. The latter of these is completely described by the octahedral splitting (10D_q_) in an O_h_ point group. The LFT Hamiltonian is then given in eq 2.^25^ The elements from top to bottom and left to right are in the following order: ^3^A_2g_, ^3^T_2g_ (t_2g_^5^e_g_^3^), ^3^T_1g_ (t_2g_^5^e_g_^3^), and ^3^T_1g_ (t_2g_^4^e_g_^4^) for the corresponding bra and ket parts of the integrals in eq 2. The off-diagonal terms represent the connection between the two ^3^T_1g_ terms
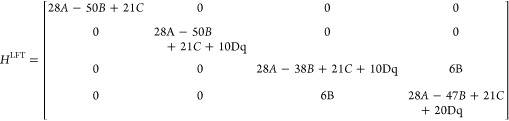
2

It is easily seen that the Hamiltonian satisfies the linear dependence on the parameter space described by p = {*A*, *B*, *C*, 10Dq},^14,49^ as stated by eq 1. The unique nonzero elements of eq 2 can be given as follows
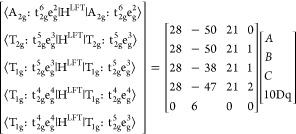
3

The quantity
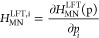
4is the first derivative of the ligand field CI matrix element M,N with respect to parameter i. We note in passing that the repulsion parameters are only well defined for *N* > 1. Furthermore, the parameter *C* is a nonredundant parameter only if more than one spin multiplicity is considered (the index for spin is omitted here for clarity).

The solution of the linear equation system (1) can be written as
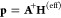
5where **A** is a matrix defined by *A*_MN,i_ = *H*_MN_^LFT,i^, A^+^ is the Moore–Penrose pseudo-inverse (Penrose, Roger. In Mathematical proceedings of the Cambridge Philosophical Society, vol. 51, no. 3. Cambridge University Press, 1955.), and **H**^eff^ is the ab initio Hamiltonian matrix. We have written it as “**H**^eff^” here since the matrix changes according to the ab initio method used. In the most elementary case, it is simply the full-CI matrix from the CASSCF.

The original AILFT procedure has the major limitation of being limited to CAS(*n*,5) spaces. This is a severe constraint because a CAS(*n*,5) is not an accurate wave function. One of the major deficiencies is that the molecular orbitals optimized in this way are too ionic since the bonding counterparts of the, generally antibonding, metal d-based molecular orbitals are not in the active space. This exaggerated ionicity will then lead to LFT parameters B and C that are too close to the free-ion values. In other words, the nephelauxetic effect will be underestimated. This has limited importance for very ionic transition-metal complexes but will significantly compromise the results for more covalent metal–ligand bonds formed between metals in higher oxidation states with “soft” ligands. The Racah parameters of the complexes may even exceed the free-ion values due to the lack of incorporation of atomic effects such as radial correlation.

Hence, the goal of this article is to develop an extension of AILFT (esAILFT) that is not limited to minimal active spaces but can be used in conjunction with more general active spaces. We will then demonstrate the importance of various active space choices on the LFT parameters including a second d-shell, the 3s3p semicore orbitals. The incorporation of ligand–metal bonding or empty ligand orbitals in the active space will be the subject of a separate study.

#### 2.1.3 Ab Initio Effective Hamiltonians

The CASCI Hamiltonian described over the d-orbital MO space (min) can be written as
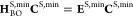
6where *S* is the spin quantum number of the block under consideration, **H**_BO_ is the Born–Oppenheimer Hamiltonian, **C** are the configuration interaction (CI) coefficients to the many-electron configurational state functions (CSFs), and **E** are the corresponding energies. As described in relation (1), the two-electron integrals are approximated within the LFT model by the relevant Slater–Condon/Racah parameters populating the parameter vector p. In the presence of a symmetry-lowering ligand field, the active orbitals deviate from their behavior from the spherically symmetric case. For instance, in the presence of an octahedral ligand, they split into t_2g_ and e_g_ sets. When the system is mostly ionic, the LFT Hamiltonian describes the CASCI Hamiltonian quite well.

However, there are a few limitations to this minimal d-orbital CASCI approach. First of all, it does not allow for the incorporation of additional metal orbitals that are important for quantitative accuracy, e.g., a second metal d-shell for the description of radial correlation or the filled semicore ns- and np-orbitals that interact strongly with the nd-shell in question (*n* = 3,4,5···). Second, the minimal active space does not allow for a balanced description of metal–ligand bonding since bonding/antibonding pairs cannot be incorporated, rather only the member that is primarily metal-based. This limits the achievable accuracy for highly covalent systems (e.g., higher oxidation state metal-ions with soft ligands) or systems with pronounced backbonding (lower oxidation state metal-ions with ligands incorporating low-lying unoccupied MOs).^6^ Third, the CASCI wave function does not incorporate dynamic electron correlation.

The latter limitation regarding the dynamic correlation has been addressed by several proposed modifications to the AILFT approach. One of the more straightforward approaches here has been to use second-order MRPT, in particular, the implementation known as NEVPT2.^2−4^ The approach in this case is to use the wave functions obtained at the CASSCF level and transform them with the NEVPT2 energies to get an effective correction term for the CASCI Hamiltonian
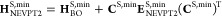
7where **E**_NEVPT2_ and **H**_NEVPT2_ are the energies obtained at the NEVPT2 level and the subsequently corrected Hamiltonian. The NEVPT2 Hamiltonian leads to a better agreement with experimental values; however, there is also an increase in the root-mean-square deviation (RMSD) of the fit between the LFT and AI-effective Hamiltonian.^55^

An alternative approach to incorporate dynamical correlation called DCD-CAS(2) has also been recently discussed which shows improvement in the prediction of LFT parameters^35−37,50^ compared to NEVPT2. The equation of the effective Hamiltonian is derived in an analogous way
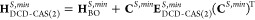
8where **E**_DCD-CAS(2)_ and **H**_DCD-CAS(2)_ are the energies obtained at the DCD-CAS(2) level and the subsequently corrected Hamiltonian. The most straightforward way to address the first two shortcomings mentioned above is to increase the size of the active space by including some of the orbitals from space internal or external with respect to the minimal active space and their corresponding electrons. While this approach presents a way to improve the physics captured at the ab initio level, the dimensionality of the resulting extended CASCI Hamiltonian is necessarily larger than the ligand field Hamiltonian matrix. Hence, it is not a priori clear how to arrive at a successful and unique mapping procedure that would allow one to extract the ligand field parameters. Our solution to this problem and its implementation into a development version of the ORCA program package^43−46,48^ will be discussed below.

### 2.2 Partitioned Hamiltonian

In order to approach the problem of reformulating AILFT in extended active spaces, we resort to partitioning theory as it is briefly described below.^41,42^

#### 2.2.1 Partitioning

The method of partitioning given by Löwdin^38−40^ presents one approach for building effective Hamiltonians. In this case, the Hilbert space is divided into a model (A) and an outer (B) space. The time-independent Schrödinger equation can be rewritten as
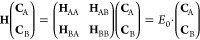
9where *E*_0_ is assumed to be the ground state of the **H**_AA_ matrix (equivalent to **E**^*S*,ext^ ≈ *E*_0_ in eq 6) This can be rewritten to eliminate **C**_B_
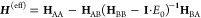
10

This type of effective Hamiltonian is essentially the sum of a Hamiltonian truncated to the model space H_AA_ and a dressing matrix  that captures the effect of the space B on A. The inverse exists when E_0_ is well separated from the energies of **H**_BB_. Diagonalization of *H*^(eff)^ gives **C**_A_ which is the projection of the exact eigenstate with energy *E*. This method acts as a useful way to build an effective Hamiltonian when the full Hamiltonian is already known, as is the case for our application.

The assumption that *E*^(0)^ is well approximated by the ground state of the A space introduces a bias toward this ground state. As it has been shown in the case of the DCD-CAS method,^35−37,50^ this introduces a correction term in the Hamiltonian expression, as presented in eq 11. Capital indices denote many electron quantities, where the indices I, J are used for the model space CSF and the indices K, L are used for an outer space CSF. H with indices in subscript denotes a single element of H from eq 9
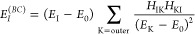
11
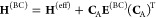
12where *E*_I_^BC^ is the bias correction to the energy of the I-th eigenvalue of **H**^(eff)^. **C**_A_ is known from **C**^S,min^. **H**^(BC)^ is the bias-corrected and partitioned Hamiltonian which will be the **H**^(eff)^ to be used in eq 5.

## 3 Implementation

### 3.1 Orbital Space

The first step in any AILFT procedure is to establish a correspondence between ab initio MOs and the fictitious d-orbitals used in LFT. To this end, we make use of the fact that the active orbitals form a unitarily invariant subspace. Consequently, we can apply any suitable unitary transformation in order to bring the active orbitals into a form that is suitable for AILFT extraction. There exist many possible ways to ensure this correspondence when dealing with the minimal space CASSCF such as the Gram-Schmidt orthonormalization of the active orbitals with respect to the remaining MOs or the diagonalization of the *L*_z_ operator over the active MOs. When dealing with a more general orbital space for a single magnetic center, the following protocol is applied for the construction of the orbitals.

In a nutshell, the calculation starts by solving the CASSCF problem in the extended active space. This, in general, results in a set of active space orbitals that do not have a clear division into metal-d- or -f-based MOs and other correlating MOs. Thus, the first step of the procedure is to localize the active space orbitals using an Augmented Hessian Foster Boys^15^ (AHFB) algorithm. This leads to the identification of metal-based active MOs. The second step in the procedure consists of diagonalizing the orbital angular momentum operator  over the now-identified d-like MOs. This produces MOs that are suitable for AILFT extraction. After some experimentation, we decided to diagonalize the sub-block of the CASSCF Fock operator corresponding to the outer space orbitals. This way, the outer space orbitals are canonicalized. The outer step canonicalization is recommended for the identification of the external space but is not necessary for our treatment. We use these orbitals in the next section to build the CI matrix H. Thus, in summary, a two-step procedure is used to divide the active orbitals into two spaces: (1) a 5 (7) dimensional space that consists of the metal-d-(f)-based MOs. These MOs are ordered in a standard manner and phase-matched to the fictitious d (or f) orbitals used in LFT. (2) The remaining MOs can be used directly or after a unitary transform that diagonalizes the sub-block of the active space Fock operator.

One of the advantages of performing CASSCF over an extended active space is that the resulting MOs capture the mixing of the d-orbitals with the remaining MOs in the extended space. However, performing the CASSCF calculation over the extended active space can cause a large number of possible CI roots. To keep this calculation manageable and still obtain the desired orbitals, only those CI roots are included in the extended space calculation that correspond to the possible CI roots from a minimal space CASSCF calculation. We do this by first generating lists of configurations over the d-orbitals and d + extended orbital space over the initial guess orbitals for the CASSCF. We use those configurations in the extended space that correspond to configurations in the minimal space to generate a set of initial CSFs for the extended CASSCF problem. We also perform a final check after the convergence of CASSCF and purification of orbitals to make sure that no root dominated by the outer space was added in the convergence iterations.

To be clear, at this stage, we are primarily concerned with orbital preparation. A summary of the preparation algorithm is as follows:

1. Converge orbitals for an extended CASSCF calculation using a specially built list of initial configurations (and therefore CSFs) built over the initial guess orbital space. These configurations have similar occupations on the d-orbitals as a corresponding minimal space calculation.
2. The MOs obtained this way are then localized to obtain distinct metal and ligand MOs. The  and (optional) Fock operators are diagonalized on the metal and remainder orbitals in order to obtain an orbital space where configurations can now be clearly labeled. This is possible due to the fact that the active space of a CASSF calculation is invariant against the unitary transformations of the active orbitals among themselves. It should be noted that the  and Fock operators operate on distinct orbital subspaces within the active space, and consequently, their actions commute by construction. Once such an extended preparation is complete, check the resulting roots to ensure no root dominated by the outer space was added in the calculation.
3. In the upcoming section, when we start building the effective Hamiltonian, we will be labeling and ordering the configurations on the prepared orbital space based on the occupations of the d-like orbitals and then appropriately constructing the effective Hamiltonian.

### 3.2 Construction of the Dressed Hamiltonian

The second step of the algorithm is the efficient generation of the effective Hamiltonian matrix for the fitting procedure. This involves generating the minimal space Hamiltonian (*H*_AA_) and then calculating a dressing matrix  that includes the effect of the remainder space. Given that the active space MOs are suitably prepared for a minimal number of roots in the previous step, the list of configuration state functions (CSFs) for all of the possible roots in the extended space is now built from the active orbitals.

The CSFs are classified according to how many electrons they contain in the metal d-(f)-based MOs. In our partitioning, CSFs are classified as belonging to the “A” space if they include N_el_^min^ electrons on the d-like MO space. N_el_^min^ is the number of d-electrons that corresponds to the d^*n*^ (f^*n*^) configuration that we are interested in. Hence, “A” is our many-particle target space. All other CSFs belong, by definition, to the “B″ space.
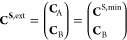
13

Using this classification, eq 10 can be used for the partitioning procedure. The easiest way to compute the partitioned Hamiltonian would involve the use of the full CI Hamiltonian over all of the roots possible in this space. However, this is clearly impractical as the number of CSFs in the large space and therefore also the number of roots can reach millions. However, this problem can be made computationally tractable by using the following treatment.

In eq 10, the dressing operator (partitioning correction) is given by
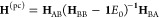
14

If the outer space is well separated in energy from the model space, then one can approximate the B-space Hamiltonian by its diagonal elements
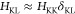
15

This results in a form analogous to eq 10
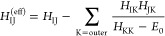
16Equation 16 requires only diagonal elements of *H*_BB_, but it still requires the H_AB_ coupling matrix. However, this changes the size of the CI matrix from  to  since N_CSF_^S,min^ does not change with the size of the extended space. However, the *H*_AB_ coupling matrix can be too large to hold in memory as the active space grows beyond a few million CSFs, and thus one needs to be careful in the precise way eq 16 is implemented. A single term in the summation of *H^(^*^pc)^ is referred to as the partial dressing (partial partitioning correction). The partial dressing is an object of the same dimension as the model space (*N*_CSF_^S,min^ × *N*_CSF_^S,min^) and can be held in memory. For the K-th term in the partial dressing, we need to generate *H*_IK_ for all I in the model space and the element *H*_KK_. Each time a term of *H*^(pc)^ is added to *H*_AA_, it can be deleted from memory, along with the smaller objects used to build it. Thus, we generate only those elements of *H*_AB_ which are required for each partial dressing, and no object larger than *H*_AA_ is ever held in memory. The matrix operations involved can be efficiently implemented using the BLAS package,^14^ leading to a highly efficient performance in terms of both time and memory.

As discussed in Section 2.2.1 the assumption *E*^S,ext^ ≈ *E*_0_ introduces a bias toward the ground state that we correct by employing eqs 11 and 12. In particular, the summation in eq 11 is merely the diagonal element of the partial dressing multiplied by a scalar (). Thus, the bias corrections to the energies **E**^(BC)^ can be calculated at the same time as **H**^(pc)^. The bias-corrected Hamiltonian **H**^(BC)^ is then calculated by using these energies after the partitioning. The resulting effective Hamiltonian is ready for the fitting procedure given by eq 3.

The individual steps in the calculation are summarized as follows:

1. Orbital preparation: Optimized CASSCF MOs are obtained by solving the CASSCF equations. We then exploit the unitary invariance of the active orbital space in order to make these orbitals suitable for the extended AILFT procedure by performing the following three steps:
  a. The active orbitals are localized which will separate metal-based d- or f- orbitals from ligand-based orbitals
  b. The metal-d- or metalf-based orbitals are canonicalized by diagonalizing the matrix representation of the angular momentum operator  and then phase-adjusted to be consistent with the ligand field orbitals. Both steps a. and b. are already present in the original AILFT procedure.
  c. The remaining outer space orbitals are transformed to diagonalize the corresponding sub-block of the Fock operator. This step is optional but, in our experience, beneficial for the partitioning procedure and the interpretation of the results.
2. Generation of the effective Hamiltonian over the ligand field manifold space, as described in eqs 14–16: The individual steps are as follows:
  a. Calculations of the actual effective Hamiltonian (eq 14)
  b. Calculation of the bias correction as in eq 12.
3. The original AILFT procedure, as given by eqs 1–3, is then used on the effective Hamiltonian.

## 4 Results and Discussion

### 4.1 Divalent and Trivalent Transition Metals

The present implementation was compared against minimal space AILFT and against experimental data when available. All calculations were performed using a development version of the ORCA program based on ORCA 6.0.^43−46,48^ The systems considered in this study are divalent and trivalent transition-metal ions described by the dominant electronic configuration of d^3^ to d^7^ from the set of 3d, 4d, and 5d transition metals (TMs). The choice of ionic metals is convenient because it allows the present study to analyze the effect of orbital extension exclusively using electron repulsion parameters B and C. This is due to the spherically symmetric nature of the free ions. While in principle C is an independently computed parameter within the present study (Values in Supporting Information), the generally accepted relationship of *C* ≈ 4*B* allows the present analysis to focus on the variations of the Racah B parameter. A separate analysis of the other AILFT parameters relevant in transition-metal complexes with reduced symmetries will be published in a subsequent study. For the inclusion of scalar relativistic effects, the X2C method^23,30^ with the matching all-electron X2C-TZVPall basis set^51^ was used.

The esAILFT method implemented is general in nature, implying that all AI-effective Hamiltonians constructed at various active space extensions are expected to improve the extracted LFT parameters, as far as the extended active space improves the description of the metal–ligand bonding, as well as capture some important dynamic correlation effects.

Our previous experience^35^ has established that the lack of any dynamic correlation and the exaggerated ionicity of the metal–ligand bonding, both of which result from the minimal CAS(*n*,5) active space, leads to Racah B-parameters which tend to be too large when compared to values obtained by fitting to experiments.^35^ Thus, improvements in the wave function are expected to be reflected in B, as will be demonstrated below.

In order to establish which factors are most important for an improved wave function, we have systematically studied the following active space extensions for the free atoms and ions (the principal quantum number of each row of the d-block would be *k* = 3, 4, 5):

a.)Addition of a (*k*+1)d-shell
b.)Addition of the (*k*+1)s- and (*k*+1)p-shells
c.)Addition of the (filled) (*k*)s- and (*k*)p-shells

**Figure 1:**
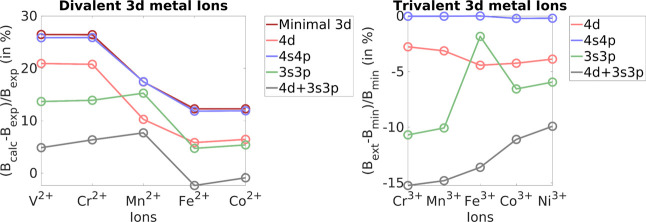
(Left) (*B*_calc_ – *B*_exp_)/*B*_exp_ (in %) for divalent 3d metal ions, where *B*_exp_ is the Racah B parameter as obtained with experiment, and *B*_calc_ is the Racah B for minimal 3d and the extensions: 4d, 4s4p, 3s3p, and 3s3p4d. (Right) (B_ext_ - *B*_min_)/*B*_min_ (in %) for trivalent 3d metal ions, where *B*_min_ is the Racah B parameter as obtained using the minimal 3d, and B_ext_ is the Racah B for extensions: 4d, 4s4p, 3s3p, and 3s3p4d.

The first set that is considered is that of 3d divalent metal ions (Figure 1: left, Table 1). These are particularly useful for comparison as the experimental values for these ions in the gas phase are readily available.^16,26,29,49^ However, the experimental fits are limited by the fact that they require precise assignment of the observed line spectra. References (26, 29, and 49) did not contain data for all the 3d trivalent ions which limits the comparison to experiment to some extent. However, as will become evident below, it is still very informative to reference the extended space AILFT results to the minimum space (original) AILFT. The latter uniformly overestimates the electron–electron repulsion parameters, and it is very interesting to study to which extent the additional orbitals in the active space alleviate this problem. The minimal AILFT was used as a reference for 4d and 5d transition metals for a similar reason. The experiment and minimal AILFT calculations are expected to represent the lower and upper bounds of the esAILFT calculations, respectively. The inclusion of extended active spaces brings the results closer to the experiment because they reduce the B value.

**Table 1:** Racah B for Divalent 3d Metal Ions (in cm^–1^)^a^

	absolute values					
	fit to experiment^26,29,49^	minimal (3d)	3d + 4d	3d + 4s4p	3d + 3s3p	3d + 3s3p + 4d
V^2+^	766	968.7	926.3	964	870.7	803.2
Cr^2+^	830	1049.3	1002.4	1044.8	945.4	882.7
Mn^2+^	960	1127.3	1058.5	1127.6	1106.5	1033.8
Fe^2+^	1058	1187.8	1119.7	1183.2	1108	1033
Co^2+^	1115	1251.9	1186.8	1247.6	1175	1105

percentage change in calculated value (reference: fit to experiment)					
	minimal (3d)	3d + 4d	3d + 4s4p	3d + 3s3p	3d + 3s3p + 4d
		(a)	(b)	(c)	(d)
V^2+^	26.5	20.9	25.8	13.7	4.9
Cr^2+^	26.4	20.8	25.9	13.9	6.3
Mn^2+^	17.4	10.3	17.5	15.3	7.7
Fe^2+^	12.3	5.8	11.8	4.7	–2.4
Co^2+^	12.3	6.4	11.9	5.4	–0.9

a Experimental values are as reported in refs (26, 29, and 49).

Fortunately, for the divalent ions of the 3d series, the experimental data are fairly complete such that a direct comparison is possible. As shown in Table 1, the original AILFT values overestimate the experimental values by about 12–26%. Curiously, the overestimation is larger for the less electronically crowded early transition metals (with fewer than five electrons in the d-space) and is reduced systematically for the later transition metals. This is a trend that appears to be counterintuitive to us and for which we do not have a concise explanation.

**Figure 2:**
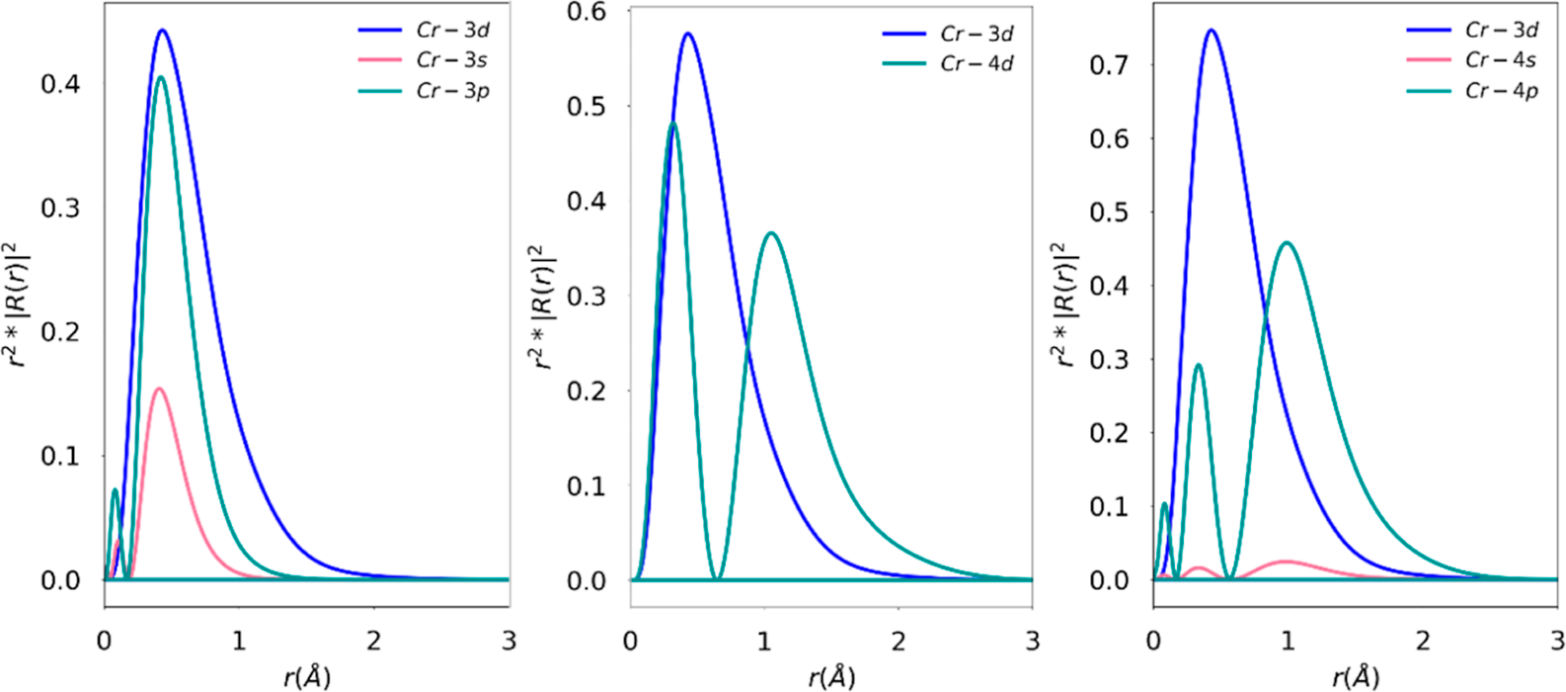
Radial function plots for Cr^2+^ for the 3s3p, 4d, and 4s4p extensions (left to right). *R*_3d_(*r*) is the radial function for the Cr-3d (blue) curve, and *R*_ext_(*r*) is the radial distribution of the external (ext = Cr 3s, 3p, 4d, 4s, 4p) orbital space (green and red curves).

Inclusion of a second d-shell reduces this error by about 5%, which should be attributed to a radial correlation effect, which is dynamic in nature. While the inclusion of a 4sp shell does not seem to be beneficial, inclusion of the semicore 3sp shell is even more effective in reducing the overestimation of the original LFT. Since the 3d and 3sp shells have similar extents (Figure 2), this should probably be interpreted as an angular correlation effect. Finally, including both the 3sp and 4d shells in the calculations impressively reduces the error to around 5% or less (except 7.7% for Mn). The contributions for the 3sp and 4d shells are approximately but not perfectly additive, and hence, as long as computational resources allow, we would recommend to include both sets of additional shells in the calculations.

In Figure 2, we emphasize the spatial relationships between the 3sp, 3d, and 4d shells. The effect of extension of the various active spaces can also be understood using the radial distributions of 3d and the various sets of extended orbitals. A greater overlap of the radial distribution leads to a more significant intershell electron–electron repulsion. The higher the overlap of the radial wave functions of two shells, the more the electrons occupying them are forced into the same region of space, and thus, the larger the electron–electron interactions will be and the larger the propensity to “escape” out of the common interaction region.

For the case of Cr^2+^, this is shown in Figure 2, where *R*_3d_(*r*) and *R*_ext_(*r*) are the radial functions for the 3d orbital and an external orbital. One may define a radial overlap between the curves in Figure 2 as the ratio of the area under two curves: the first is the area under the curve for the minimum of radial distribution functions of 3d () and an “external” orbital (), and the second is the area under the curve of the radial distribution of 3d (). This way, the overlaps are normalized to area under the () curve calculated for a finite distance (here, taken to be 0 to 3 Å from the nucleus). The 3s3p shell has a 78% overlap (20% for 3s + 59% for 3p), 4d has a 59% overlap, and 4s4p (4% for 4s + 42% for 4p) has a 46% overlap. We can see at a glance from the radial distribution plot that the 4s4p shell has the smallest possible overlap with the 3d space of the Cr^2+^ ion. This argument is analogous to the energy differences shown in Table 2. The calculations show that the extension of an active space to the 4d and 4s4p orbital spaces for the divalent transition metals gives the greatest improvement to the more than half-filled configurations of ions such as Fe^2+^ and Co^2+^.

**Table 2:** Energies of Spectroscopic Terms at CASSCF of Various Active Spaces Compared to Experiment in cm^–1^ for Cr^3+^ (3d^3^)^a^

active space	*E*(^4^P)	*E*(^2^G)	*E*(^2^P)	*E*(^2^H)	*E*(^2^F)
CAS(11,14) (3s3p3d4d)	14,351 (−3019)	16,410 (−1221)	19,526 (−3895)	23,964 (543)	36,922 (−3870)
CAS(11,9) (3s3p3d)	14,983 (−2387)	17,225 (−406)	20,445 (−2976)	25,230 (1809)	38,815 (−1977)
CAS(3,10) (3d4d)	17,040 (−330)	17,148 (−483)	22,863 (−558)	22,753 (−668)	39,710 (−1082)
CAS(3,5) (3d)	17,370 (0)	17,631 (0)	23,421 (0)	23,421 (0)	40,792 (0)
Experimental^34^	13,758 (−3612)	14,700 (−2931)	18,919 (−4502)	20,658 (−2763)	33,899 (−6893)

a Values in parentheses are deviations from CAS (3,5) calculations.

A closer look at the spectroscopic transition energies of Cr^3+^ (Table 3) reveals that, as expected, the expansion of the active space causes the values to approach the values given by the NIST database.^34^ Of particular interest are the ^2^H and ^2^P term energies, which show an accidental degeneracy when the active space is restricted to CAS(3,5). This degeneracy is also predicted by the Slater–Condon theory and is an artifact of the restriction of the active space. The expansion to the 4d space reduces the energies of these two levels and causes a small splitting already lowering the energy of ^2^H compared to ^2^P by about 110 cm^–1^, which is opposite to the experiment. The major and corrective part of the splitting, however, occurs due to the inclusion of the 3s3p orbitals. This introduces a much larger splitting of 4785 cm^–1^, which once again brings ^2^P lower than ^2^H, restoring the correct ordering of the energies. One can clearly see how the incorporation of these extensions is needed to capture the essential physics that exists in systems like these.

**Table 3:** Racah B for Trivalent 3d Metal Ions (in cm^–1^)^a^

	absolute					
	fit to experiment^26,29,49^	minimal (3d)	3d + 4d	3d + 4s4p	3d + 3s3p	3d + 3s3p + 4d
Cr^3+^	1030	1158.1	1126	1157.8	1034.3	981.6
Mn^3+^	1140	1234.5	1195.8	1234.2	1110.2	1051.7
Fe^3+^		1307.3	1249.4	1307.3	1283.3	1129.4
Co^3+^		1365.9	1307.9	1362.9	1276.3	1214.3
Ni^3+^		1427.9	1372.5	1425	1343	1286.3

	percentage change in calculated value (reference: minimal)				
	fit to experiment^26,29,49^	3d + 4d	3d + 4s4p	3d + 3s3p	3d + 3s3p + 4d
Cr^3+^	–11.1	–2.8	0	–10.7	–15.2
Mn^3+^	–7.7	–3.1	0	–10.1	–14.8
Fe^3+^		–4.4	0	–1.8	–13.6
Co^3+^		–4.2	–0.2	–6.6	–11.1
Ni^3+^		–3.9	–0.2	–5.9	–9.9

a Experimental values are as reported in refs (26, 29, and 49).

In the case of trivalent 3d metals (Figure 1: right, Table 3), the experimental values of Racah B have an average percentage deviation with respect to the minimal space calculations of about −9.4%. The contributions of the external 4d shell and internal 3s3p shell are comparable with the average percentage change of −3.7% and −7.0%, respectively, compared to the minimal space calculations. Once again, the energy separation of the external 4s4p orbitals leads to an average percentage change of only −0.1% compared with the minimal space. As seen in the case of divalent systems, the 4s4p orbitals have once again negligible influence on the Racah B parameters of these ions. The inclusion of 3s3p and 4d orbitals at the same time gives the highest percentage change of −12.9%.

**Figure 3:**
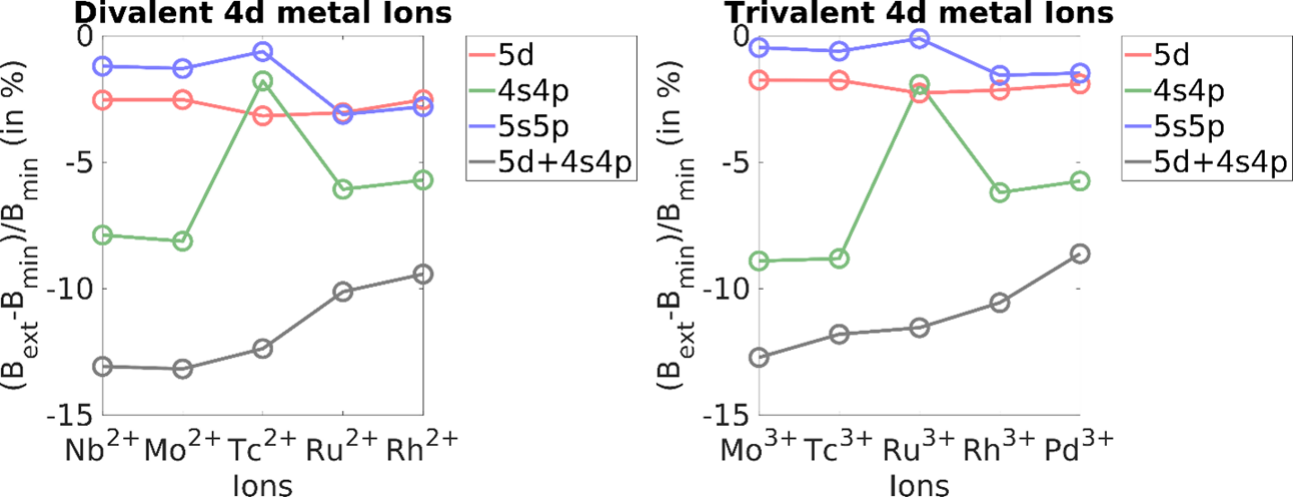
(*B*_ext_ – *B*_min_)/*B*_min_ (in %) for divalent and trivalent 4d metal ions. *B*_min_ is the Racah B parameter, as obtained using the minimal 4d space, and *B*_ext_ is the Racah B parameter for extensions to 5d, 5s5p, and 4s4p spaces.

The next set of systems considered is the 4d metal ions (Figure 3, Table 4). In the divalent case, the average percentage change for the Racah B parameter compared to the minimal 4d space was −2.7%, −1.8%, −5.9%, and −11.6% for the extension to 5d, 5s5p, 4s4p, and 4s4p+5d orbital spaces, respectively. With the trivalent 4d ions, the average percentage change for the Racah B parameter of −2.0%, −0.9%, −6.3%, and −11.0% was calculated for the same orbital extensions with respect to the minimal 4d space. We see that the three orbital extensions are more comparable in these cases and that the relative improvement due to the internal 4s4p shell is always higher. The more diffuse 4d orbitals seem to benefit less from further orbital extensions as the electrons are already quite well separated and experience low repulsion.

**Table 4:** Racah B for Divalent and Trivalent 4d Metal Ions (in cm^–1^)

	absolute value					percentage change in calculated value (reference: minimal 4d)			
	minimal (4d)	4d + 5d	4d + 5s5p	4d + 4s4p	4d + 4s4p + 5d	4d + 5d	4d + 5s5p	4d + 4s4p	4d + 4s4p + 5d
Nb^2+^	708.4	690.4	699.9	652.6	615.8	–2.5	–1.2	–7.9	–13.1
Mo^2+^	764.7	745.4	754.8	702.6	664	–2.5	–1.3	–8.1	–13.2
Tc^2+^	818.3	792.4	813.2	803.7	717.1	–3.2	–0.6	–1.8	–12.4
Ru^2+^	861.8	835.6	835	809.5	774.6	–3	–3.1	–6.1	–10.1
Rh^2+^	906.6	883.7	881.2	854.9	821.2	–2.5	–2.8	–5.7	–9.4
Mo^3+^	817.8	803.5	814	745	713.8	–1.7	–0.5	–8.9	–12.7
Tc^3+^	868.8	853.5	863.5	792.3	766.3	–1.8	–0.6	–8.8	–11.8
Ru^3+^	918.6	897.8	917.6	901	812.6	–2.3	–0.1	–1.9	–11.5
Rh^3+^	960.1	939.5	945.1	900.6	858.8	–2.1	–1.6	–6.2	–10.6
Pd^3+^	1003	983.9	988.2	945.4	916.6	–1.9	–1.5	–5.7	–8.6

**Figure 4:**
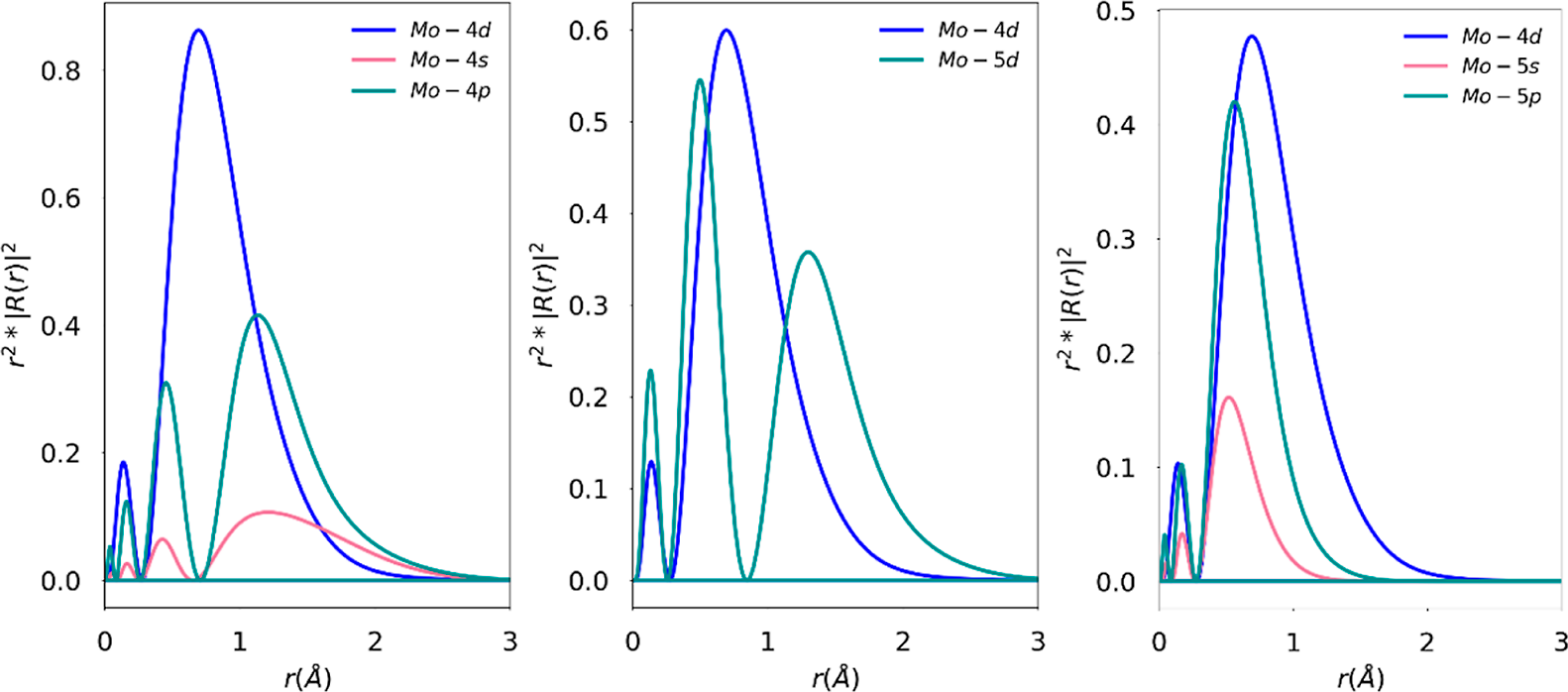
Radial correlation plots for Mo^3+^ for the 4s4p, 5d, and 5s5p extensions (left to right). *R*_4d_(r) is the radial function for the Mo 4d (blue) curve, and *R*_ext_(r) is the radial distribution of the external (ext = Mo 4s, 4p, 5d, 5s, 5p) orbital space (green and red curves).

The radial distribution plots for the 4d and the extended orbitals are shown for Mo^3+^ in Figure 4. An overlap percentage of the extended orbitals with reference to the 4d orbitals can be defined analogous to the 3d case and normalized to the area under Mo-4d for 0–3 Å from the nucleus. The overlap percentages are 57% for the 5d, 75% for the 4s4p (20% 4s + 55% 4p), and 62% for the 5s5p (17% 5s + 45% 5p) orbitals. The relative overlap is predictive of the higher contribution of the 4s4p shell to the improvement in Racah B of Mo^3+^, as seen in Table 4. This is consistent with the argument for Cr^2+^ seen earlier for the 3d case. However, we see for Mo^3+^ that the external 5s5p and 5d shells have similar contributions toward the improvement of Racah B, unlike Cr^2+^ where 4d has a dominant contribution to Racah B. This highlights the fact that all three orbital spaces need to be considered for 4d metals, as the qualitative difference between the three spaces is not as distinct as the 3d case.

**Figure 5:**
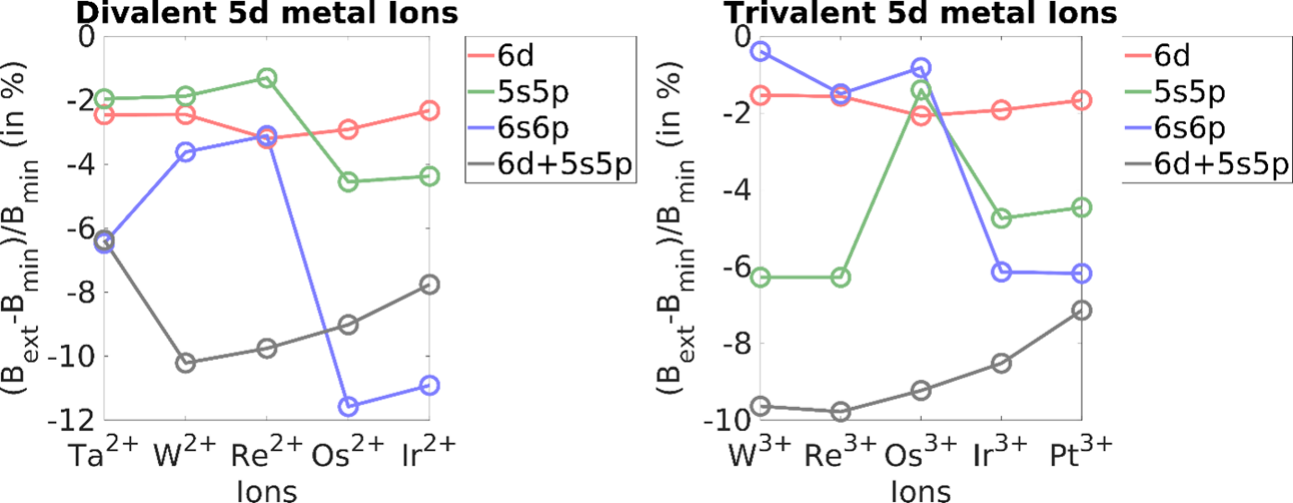
(*B*_ext_ – *B*_min_)/*B*_min_ (in %) for divalent and trivalent 5d metal ions. *B*_min_ is the Racah B parameter, as obtained using the minimal 5d space, and *B*_ext_ is the Racah B parameter for extensions to 6d, 6s6p, and 5s5p spaces.

For the 5d metal ions (Figure 5, Table 5), the divalent case has an average percentage change of −2.7%, −7.1%, and −2.8% for extensions to 6d, 6s6p, and 5s5p orbital spaces with respect to the minimal 5d space, while the trivalent case has an average percentage change of about −1.8%, −4.6%, and −8.9%, respectively. Being the largest of the d orbitals considered, the lowest improvements to the orbital space extension are present in this case. The lower energy gaps of the 6s6p orbitals with respect to the 5d shell seem to cause greater improvement in the Racah B values. Notably, Os^2+^ and Ir^2+^ have a greater contribution from the 6s6p shell unlike the other systems in the data set. Thus, the systematic use of an internal 5s5p + external 6d shell in the benchmark is not able to exceed the 6s6p contributions here. This is merely a reflection of the choice of orbital extension criteria of our present benchmark.

**Table 5:** Racah B for Divalent and Trivalent 5d Metal Ions (in cm^–1^)

	absolute value					percentage change in calculated value (reference: minimal 5d)			
	minimal (5d)	5d + 6d	5d + 6s6p	5d + 5s5p	5d + 5s5p + 6d	5d + 6d	5d + 6s6p	5d + 5s5p	5d + 5s5p + 6d
Ta^2+^	668.9	652.5	625.6	655.8	626.3	–2.5	–6.5	–2	–6.4
W^2+^	715.9	698.4	690	702.5	642.8	–2.4	–3.6	–1.9	–10.2
Re^2+^	759.7	735.4	736.1	749.8	685.6	–3.2	–3.1	–1.3	–9.8
Os^2+^	793.1	770	701.3	757	721.6	–2.9	–11.6	–4.6	–9.0
Ir^2+^	827.7	808.5	737.4	791.5	763.5	–2.3	–10.9	–4.4	–7.8
W^3+^	766.1	754.3	763.1	718	692.3	–1.5	–0.4	–6.3	–9.6
Re^3+^	805.9	793.3	793.8	755.3	727.1	–1.6	–1.5	–6.3	–9.8
Os^3+^	844.3	826.8	837.4	832.5	766.4	–2.1	–0.8	–1.4	–9.2
Ir^3+^	874.8	858	821.1	833.3	800.3	–1.9	–6.1	–4.7	–8.5
Pt^3+^	906.6	891.5	850.6	866.2	841.9	–1.7	–6.2	–4.5	–7.1

## 5 Conclusions

In this work, we have introduced a generalized framework called esAILFT with which extended active space can be incorporated into the framework of AILFT. In particular, a new effective Hamiltonian AI for AILFT was derived on the basis of the partitioning method and a subsequent bias correction. The esAILFT method allows for the extraction of AILFT parameters from a CASSCF calculation of arbitrary sizes. This development addresses one of the major limitations of the current AILFT protocol.

As a first application of the esAILFT method, we focused on the gas-phase, transition-metal ions with spherical symmetry, which allowed monitoring of a single variable in the Racah B parameter for transition metals as a function of various extended spaces. While all orbital space extensions are in principle expected to improve parameter extraction, we benchmarked the parameter extraction using esAILFT over three key spaces: (*k*+1)d-shell, (*k*+1)s- and (*k*+1)p-shells, and (*k*)s- and (*k*)p-shells (with *k* = 3, 4, 5 is the principal quantum number for each row of the d-block). The benchmarking was performed for the case of 3d, 4d, and 5d transition-metal ions with a +2 and +3 charge. Overall, a clear improvement of the extracted Racah B parameter for all tested cases was observed by including additional orbitals in the active space that bring in some dynamic correlation effects. The numerical trends seen by comparing the data on the basis of minimal and extended LFT spaces were found to be consistent with an intuitive understanding of the LFT picture of the studied systems. Hence, in practice, the addition of (*k*+1)d-shell, (*k*+1)s- and (*k*+1)p-shells, as well as the (*k*)s- and (*k*)p-shells, leads to improved Racah B parameters. While these effects become less and less pronounced in 4d and 5d studied systems owing to the electron repulsion decrease in the sequence, they are not negligible, highlighting the necessity of using proper AI-effective Hamiltonians in the AILFT framework.

Computationally speaking, the main bottleneck of the present methodology is the solution of the extended space CASSCF/CASCI problem. The setup of the effective Hamiltonian and the solution of the AILFT equations are, comparatively speaking, negligible in terms of the timings. This provides a positive outlook for the possibility to combine the extended AILFT method with approximate full-CI methods such as the iterative configuration interaction (ICE)^20,21^ or other large-scale approximate full-CI methods such as the density matrix renormalization group (DMRG).^17−19,24,54^

Further analysis of the method in the framework of transition-metal complexes with increasing degrees of covalency and explicit inclusion of ligand orbitals in the extended active space will be the subject of a follow-up study. Our efforts are ongoing to further expand our AILFT implementation beyond earlier implementation involving a single shell in an effort to provide streamlined and automatic parametrization for a variety of AI Hamiltonians.

## Supporting Information Available

Further details such as the comparison of the 3d divalent ions to the minimal d-space calculation and sample ORCA inputs for the present implementation (PDF)
