# Supplementary material for: Extended Active Space Ab Initio Ligand Field Theory: Applications to Transition-Metal Ions
Source: Inorg Chem. 2024 Dec 19;63(52):24672–84. doi: 10.1021/acs.inorgchem.4c03893 (PMC11688661; doi:10.1021/acs.inorgchem.4c03893)
Supplement: Supplementary file 1 — ic4c03893_si_001.pdf [file ic4c03893_si_001.pdf]

Supporting Information

An Extended Active Space Ab initio Ligand Field Theory: Applications to transition metal ions.

Shashank V. Rao[1], Dimitrios Maganas[1], Kantharuban Sivalingam[1], Mihail Atanasov[12] and Frank Neese*[1]

[1]Max-Planck-Institut für Kohlenforschung, Kaiser-Wilhelm-Platz 1, 45470 Mülheim an der Ruhr, Germany

[2]Institute of General and Inorganic Chemistry, Bulgarian Academy of Sciences, Akad. Georgi Bontchev Street 11, 1113 Sofia, Bulgaria

* neese@kofo.mpg.de

Table of Contents

1. 3d divalent ions Compared to Minimal Space S2

2. Data for Racah C parameters for systems considered S2

3. Representative Inputs S4

A. V2p_min.inp...S4

B. V2p_3s3p.inp...S4

C. V2p_3s3plm.inp S5

D. V2p_largereglm.inp..S6

1. 3d divalent ions Compared to Minimal Space

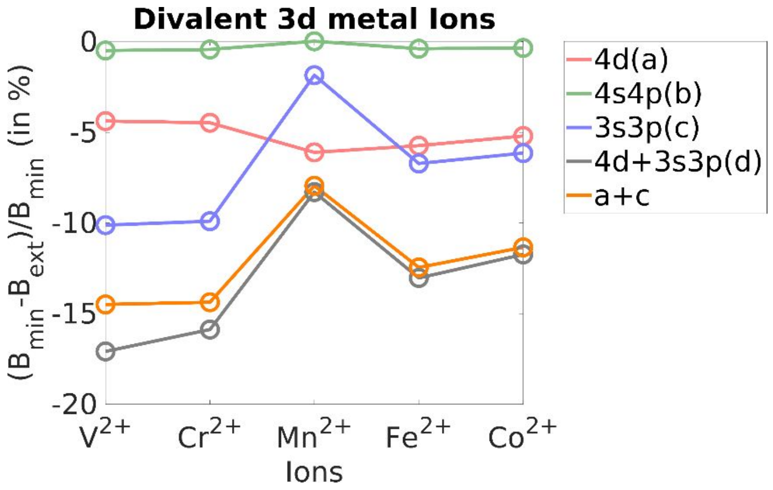

Figure S1. Shows $(B_{ext} - B_{min})/ B_{min}$ (in %) for divalent 3d metal ions where B_{min} is the Racah B parameter as obtained using the minimal and B_{ext} is the Racah B for extensions: 4d, 4s4p, 3s3p, and 3s3p4d. (a)+(c) is the sum of the changes due to individual active space extensions

2. Data for Racah C parameters for systems considered

	Absolute Values					
	Fit to Experiment	Minimal (3d)	3d + 4d	3d + 4s4p	3d + 3s3p	3d + 3s3p + 4d
V^{2+}	2855	3587	3324.3	3463.8	3343	3041
Cr^{2+}	3430	3884.6	3611.9	3769.6	3529.4	3250.1
Mn^{2+}	3325	4174.3	3924.8	4052.9	3529.4	3279.2
Fe^{2+}	3901	4389.7	4194.2	4283.8	3852.2	3691.6
Co2+	4366	4621	4463.1	4517.1	4037.3	3952.5

	Absolute					
	Experiment[25]	Minimal (3d)	3d + 4d	3d + 4s4p	3d + 3s3p	3d +3s3p + 4d
Cr^{3+}	3850	4333.3	4119.9	4323.9	4013	3781.9
Mn^{3+}	3675	4617.1	4378	4608.8	4191.8	3986.7
Fe^{3+}	-	4883.9	4669.4	4875.8	4139.2	4181.3
Co^{3+}	-	5092.2	4928.8	5006.2	4476.8	4348.7
Ni^{3+}	-	5315.2	5180	5228.7	4653.7	4594

	Absolute Value				
	Minimal (4d)	4d + 5d	4d + 5s5p	4d + 4s4p	4d +4s4p + 5d
Nb^{2+}	2810.1	2674.3	2539.2	2656.5	2499.8
Mo^{2+}	3042.6	2905	2861.9	2816.3	2680.9
Tc^{2+}	3264.1	3138.8	3124.6	2857.6	2849.1

Ru^{2+}	3435.6	3331.7	3369.1	3080.5	2979.4
Rh^{2+}	3615.3	3521.9	3560.7	3220	3143.3
Mo^{3+}	3293	3181.5	3150.1	3088.9	2958.2
Tc^{3+}	3504.1	3390.9	3393.4	3231.2	3111.5
Ru^{3+}	3708.4	3604.1	3603.4	3232.4	3263.1
Rh^{3+}	3871.7	3780	3810.7	3460.4	3402.5
Pd^{3+}	4043.1	3959.5	3989.6	3590.9	3521.7

	Absolute Value				
	Minimal (5d)	5d + 6d	5d + 6s6p	5d + 5s5p	5d + 5s5p + 6d
Ta^{2+}	2673.7	2547.8	1655.3	2690.4	2417.2
W^{2+}	2879.1	2754.8	2367.8	2885.1	2611
Re^{2+}	3070.3	2963.5	2787.8	2789.4	2767.6
Os^{2+}	3210.8	3128.8	3169.9	2968.5	2897.9
Ir^{2+}	3357.8	3286.3	3373.6	3090.8	3024.2
W^{3+}	3124	3031.1	2890.4	2975.6	2873.4
Re^{3+}	3297.6	3202.5	3056.8	3101.7	3008.2
Os^{3+}	3465.4	3380.3	3279.6	3134	3141.9
Ir^{3+}	3592	3519.7	3554.7	3311	3254.5
Pt^{3+}	3726.3	3660.5	3721.5	3420.4	3360.3

3. Representative Inputs

A. V2p_min.inp

```
!x2c x2c-TZVPall AUTOAUX

%maxcore 4000

%casscf
        # run the AILFT with the minimal active space
        actorbs dorbs

        # definition of the active space, multiplicity and number of roots
        nel 3
        norb 5
        mult 4,2
        nroots 10,40
        trafostep RI

        # optional properties such as SOC splitting and g-matrix
        rel
        dosoc false
        gtensor false
        end
end

# geometry block
*xyz 2 4
V    0.0        0.0        0.0
*
# enable picture change
%rel
        picturechange true
end
```

B. V2p_3s3p.inp

```
!x2c x2c-TZVPall AUTOAUX moread
%moinp "V2p_min.gbw"  # contains the  converged orbitals with the minimal active space


%maxcore 4000


%casscf
        # definition of the extended active space
        norb 9
        nel 11
        mult 4,2
        nroots 10,40
        trafostep RI
end

*xyz 2 4
V    0.0        0.0        0.0
*
# enable picture change
%rel
        picturechange true
end
```

C. V2p_3s3plm.inp

```
!x2c x2c-TZVPall AUTOAUX moread
%moinp "V2p_3s3p.gbw" # converged casscf orbitals with the extended active space

%maxcore 4000

%casscf
        # run the esAILFT
        actorbs lmorbs
        ailft
            AILFT_SkipOrbOpt true
            NOrbInternal 4 # no of orbitals in the extended space to be labelled as internal
            NOrbExternal 0 # no of orbitals in the extended space to be labelled as external
            MinElectrons 3 # no of electrons in the active space after external + internal are cut
        end

        # definition of the active space, multiplicity and number of roots
        norb 9
        nel 11
        mult 4,2
        nroots 10,40
        trafostep RI


        rel
        dosoc false
        gtensor false
        end
end

*xyz 2 4
V     0.0          0.0          0.0
*
%rel
        picturechange true
end
```

D. V2p_largereglm.inp

```
!x2c x2c-TZVPall AUTOAUX moread
%moinp "V2p_largereg.gbw"  # converged casscf orbitals with the extended active space

%maxcore 4000

%casscf
        # run the esAILFT
        actorbs lmorbs
        ailft
            AILFT_SkipOrbOpt true
            NOrbInternal 4 # no of orbitals in the extended space to be labelled as internal
            NOrbExternal 5 # no of orbitals in the extended space to be labelled as external
            MinElectrons 3 # no of electrons in the active space after external + internal are cut
        end

        # definition of the active space, multiplicity and number of roots
        norb 14
        nel 11
        mult 4,2
        nroots 10,40
        trafostep RI


        rel
        dosoc false
        gtensor false
        end
end

*xyz 2 4
V     0.0          0.0          0.0
*
%rel
        picturechange true
end
```
